# Oxygen Reduction by Amide-Ligated Cobalt Complexes: Effect of Hydrogen Bond Acceptor

**DOI:** 10.3390/molecules30153274

**Published:** 2025-08-05

**Authors:** Zahra Aghaei, Adedamola A. Opalade, Victor W. Day, Timothy A. Jackson

**Affiliations:** 1 Department of Chemistry and Center for Environmentally Beneficial Catalysis, University of Kansas, Lawrence, KS 66045, USA; zahra.aghaei@ku.edu (Z.A.); aaopalade@gmail.com (A.A.O.); 2 X-Ray Crystallography Laboratory, University of Kansas, Lawrence, KS 66045, USA; victorwday@gmail.com

**Keywords:** oxygen reduction reaction, cobalt coordination complexes, secondary coordination sphere, electrocatalysis

## Abstract

The ability of earth-abundant metals to serve as catalysts for the oxygen reduction reaction is of increasing importance given the prominence of this reaction in several emerging technologies. It is now recognized that both the primary and secondary coordination environments of these catalysts can be modulated to optimize their performance. In this present work, we describe two Co^II^ complexes [Co^II^(PaPy_2_Q)](OTf) (**1**) and [Co^II^(PaPy_2_N)](OTf) (**2**) that catalyze chemical and electrochemical dioxygen reduction. Both **1** and **2** contain Co^II^ centers in a N_5_^−^ coordination environment, but **2** has a naphthyridine group that places a nitrogen atom in the secondary coordination sphere. Solid-state X-ray crystallography and solution-state spectroscopic measurements reveal that, apart from this second-sphere nitrogen in **2**, complexes **1** and **2** have essentially identical properties. Despite these similarities, **2** performs the chemical reduction of dioxygen ~10-fold more rapidly than **1**. In addition, **2** has an enhanced performance in the electrochemical reduction of dioxygen compared to **1**. Both complexes yield a significant amount of H_2_O_2_ in the chemical reduction of dioxygen (>25%). The enhanced catalytic performance of **2** is attributed to the presence of the second-sphere nitrogen atom, which might enable the efficient protonation of cobalt–oxygen intermediates formed during turnover.

## 1. Introduction

The catalytic reduction of dioxygen (O_2_) is important in energy storage technologies, fuel cells, and metal–air batteries [1]. The combination of the oxygen reduction reaction (ORR) and fuel oxidation generates an electromotive force capable of powering vehicles, electronic devices, and homes [2]. Molecular oxygen can undergo reduction via the following two distinct pathways:

O_2_ + 2H^+^ + 2e^−^ → H_2_O_2_ (1)

O_2_ + 4H^+^ + 4e^−^ → 2H_2_O (2)

The conventional process for generating hydrogen peroxide (the so-called anthraquinone process) is energy-demanding. An alternative process for H_2_O_2_ production, which is potentially more sustainable, is the electrochemical, two-electron/two-proton reduction of O_2_ to H_2_O_2_ (Equation (1)) [3]. The four-electron/four-proton reduction of dioxygen to generate water (Equation (2)) is used in fuel cells [4]. The catalytic reduction of molecular oxygen is also important in aerobic respiration in biology, where cytochrome c oxidase converts dioxygen to water [5]. Other examples of dioxygen reduction in biology include amine oxidase and galactose oxidase enzymes, which reduce O_2_ to hydrogen peroxide [6], and multi-copper oxidases that reduce O_2_ to H_2_O [7].

The selectivity of the ORR towards water or hydrogen peroxide by metal catalysts is influenced by several factors, such as the electronic and steric features of the ligand and the primary and secondary coordination spheres of the metal center. Because the coordination spheres of homogeneous metal catalysts can be more easily tuned than their heterogeneous analogues, studies of molecular catalysts have played an important role in understanding the mechanism of dioxygen reduction [8].

Currently, platinum nanoparticles are the benchmark for ORR catalysts. However, the scarcity and expense of platinum have motivated the development of cost-effective alternatives based on first-row transition metals [9,10]. Cobalt complexes are being increasingly recognized as promising ORR catalysts. For instance, Duboc et al. demonstrated that a bimetallic Co-based complex supported by a bis-thiolate N_2_S_2_-donor ligand was capable of electrochemically and chemically catalyzing the reduction of dioxygen to water, using octamethylferrocene (Me_8_Fc) as a reducing reagent and 2,6-lutidinium tetrafluoroborate (LutHBF_4_) as the proton source [11]. Although numerous studies have explored the mechanisms and activities of earth-abundant metal catalysts in reducing O_2_, there is still an incomplete understanding of how particular changes in the metal primary and secondary coordination spheres can be used to tailor catalyst performance [12,13]. Extensive research has focused on the development of Fe-based catalysts that incorporate secondary coordination sphere effects to boost catalyst performance in the ORR [14,15,16,17,18,19]. In contrast, although numerous molecular cobalt catalysts have been developed to reduce O_2_ via either the 2e^−^/2H^+^ or 4e^−^/4H^+^ pathways, only a few studies have demonstrated how secondary coordination sphere interactions influence the performance of cobalt ORR catalysts [20,21]. Studies have shown that the steric hindrance of access to the cobalt center [22] and the presence of cationic groups [23] in the second coordination sphere can play important roles in modulating ORR catalyst performance.

**Scheme 1:**
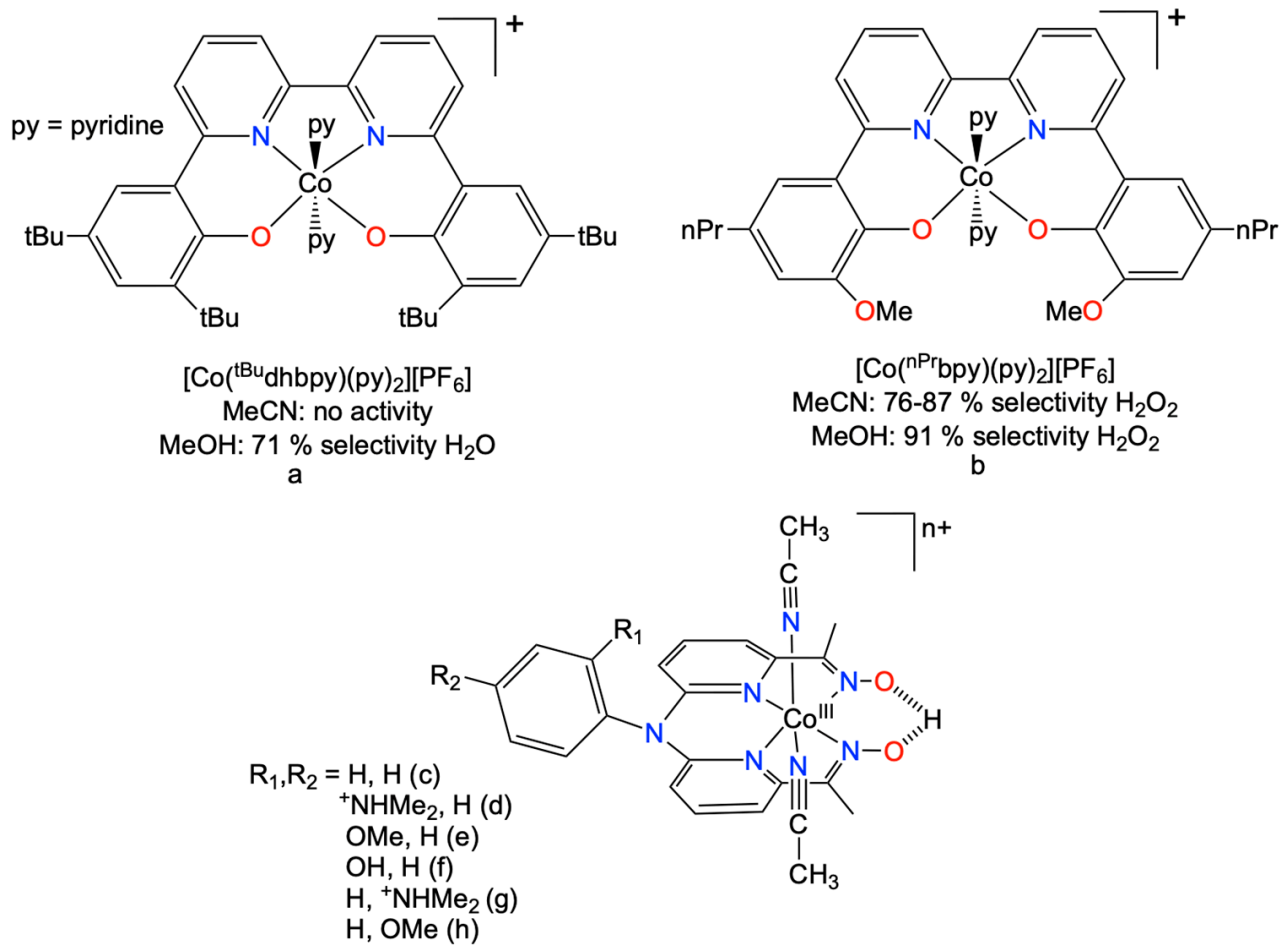
Structures of Co ORR catalysts with second-sphere Lewis bases. Top (**a**,**b**): Machan complexes based on N_2_O_2_ ligand [24]. Bottom (**c**–**h**): Paria complexes supported by bis-pyridine-bis-oxime framework [8,25].

Several recent studies have shown how the ORR activity and selectivity of cobalt catalysts can be tuned by appending Lewis bases in the secondary coordination sphere. For example, Machan reported catalytic dioxygen reduction to water in MeOH by a Co^III^ complex supported by a bipyridine-bis-phenolate (N_2_O_2_) ligand (Scheme 1a) [24]. No catalytic activity was observed in MeCN. A modified version of this catalyst with methoxy groups near the O_2_ binding site reduced dioxygen to H_2_O_2_ in MeOH and was catalytically active in MeCN (Scheme 1b) [24]. Paria and co-workers described a Co^III^ catalyst supported by a bis-pyridine-bis-oxime ligand (Scheme 1c) [8]. In phosphate buffer at pH = 4, the catalyst favored the 4e^−^/4H^+^ pathway to give H_2_O. The selectivity was altered at a higher pH, with ~75% water formation at pH = 7 and 65% H_2_O_2_ generation at pH = 11. It was proposed that the oxime moiety in the second coordination sphere of this catalyst acts as a proton shuttle [8]. More recently, Paria and co-workers expanded on this initial complex by generating a series of Co^III^ complexes using the bis-pyridine-dioxime framework but with the aryl moiety appended with different substituents (Scheme 1d–h). All complexes reduced O_2_ through the 4e^–^/4H^+^ pathway, but the complexes bearing ortho-NHMe_2_^+^ and ortho-OMe substituents (Scheme 1d,e) had turnover frequencies (TOF) increased by ~1000- and ~250-fold compared to the unsubstituted parent complex [25].

**Scheme 2:**
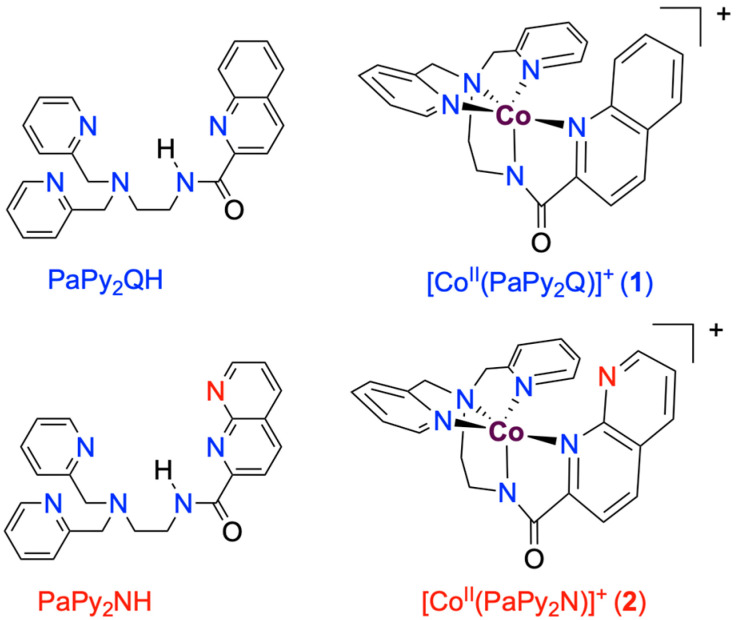
Structures of PaPy_2_QH and PaPy_2_NH ligands (**left**) and complexes **1** and **2** (**right**).

In this present study, we report two Co^II^ complexes with amide-containing, anionic N5 ligands (Scheme 2) that act as catalysts in the reduction of molecular oxygen. The Co^II^ complexes [Co^II^(PaPy_2_Q)]^+^ (**1**) and [Co^II^(PaPy_2_N)]^+^ (**2**) have identical first coordination spheres (PaPy_2_QH (N,N-Bis(2-pyridylmethyl)amine-N-ethyl-2-quinolinecarboxamide) and PaPy_2_NH (N-(2-(bis(pyridine-2-ylmethyl)amino)ethyl)-1,8-naphthyridine-2-carboxamide)). However, the PaPy_2_N ligand of complex **2** includes a naphthyridine moiety that introduces a nitrogen atom into the secondary coordination sphere.

The presence of this Lewis base gives complex **2** the ability to engage in proton delivery and/or hydrogen bonding interactions with cobalt–oxygen intermediates that might form during dioxygen reduction. A comparison of the ORR activities of these complexes, therefore, provides a probe of how a very specific modification of the secondary coordination sphere influences catalysis. Compared to **1**, complex **2** demonstrates a nearly 10-fold increase in the rate of dioxygen reduction, has an approximately 23-fold higher efficiency based on TOFi values, and exhibits more activity in the electrochemical reduction of dioxygen.

## 2. Results

### 2.1. Formation and Characterization of Complexes [Co^II^(PaPy_2_Q)](OTf) (***1***) and Na[Co^II^(PaPy_2_N)](OTf)_2_ (***2***)

**Figure 1:**
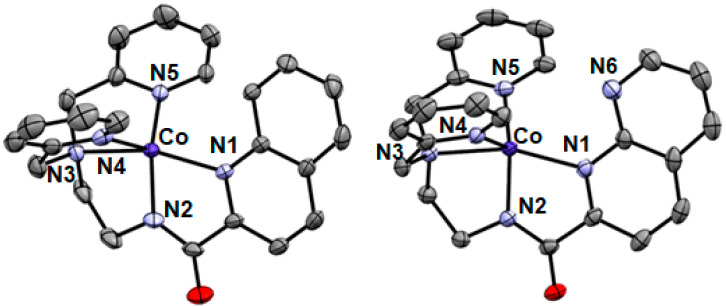
X-ray crystal structures of **1** (**left**) and **2** (**right**) showing 50% probability thermal ellipsoids. The counter ions, solvent molecules, and hydrogen atoms were removed for clarity.

Complexes **1** and **2** were prepared by adding Co(OTf)_2_ to PaPy_2_QH and PaPy_2_NH in methanol under a nitrogen atmosphere. The solid-state structures of **1** and **2** were determined by X-ray crystallography (Figure 1). Each complex has a Co^II^ center in a distorted trigonal bipyramidal environment (τ = 0.73 and 0.67 for **1** and **2**, respectively). The PaPy_2_Q and PaPy_2_N ligands are bound in a pentadentate fashion, with the pyridyl moieties giving N(pyridine)–Co–N(pyridine) angles of 118° and 114°, respectively.

For **1**, the crystal structure shows a non-coordinating triflate counter anion, with the closest Co···O distance of 5.67 Å. The unit cell of complex **2** shows Na^+^ ions from the NaOtBu used during its preparation. Two Na^+^ ions and four triflate counter anions form a bridge between separate units of **2**, with the O(amide) groups of each unit of **2** serving as ligands to Na^+^ (Supplementary Materials, Figure S1).

An overlay plot of **1** and **2** (Supplementary Materials, Figure S1) reveals essentially identical structures. Specifically, all nonhydrogen atoms superimpose with a root-mean-square deviation of 0.146 Å. The displacement of Co from the N2, N4, N5 mean plane is identical (0.36 Å) in both molecules, and the N1, N2, and N3 groupings are essentially perpendicular to the N2, N4, N5 triangle, as one would expect for a trigonal bipyramid (see Figure S1 for coordination polyhedra). A comparison of the Co–N bond lengths between **1** and **2** shows only minor differences (Table 1). For example, the Co–N1 bond in **2** (2.0806(19) Å) is slightly shorter than that of **1** (2.107(3) Å), which could reflect a difference between the naphthyridine and quinoline ligands. Indeed, the crystal structures of the [Mn^II^(PaPy_2_Q)](OTf) and [Mn^II^(PaPy_2_N)](OTf) complexes show that the Mn–N(quinoline) bond in **1** is longer than the Mn–N(naphthyridine) bond in **2** (2.357(2) and 2.228(3) Å, respectively) [26]. The Co–N2 bond of **1**, which involves the amide nitrogen, is slightly shorter than that of **2**, which might reflect the involvement of the O(amide) group in **2** with the Na^+^ ions (Supplementary Materials, Figure S1). The other Co–N2, Co–N3, and Co–N4 distances are quite similar for **1** and **2** (Table 1). Given the very similar Co–N bond lengths between **1** and **2**, the biggest difference is in the presence of N6 in the naphthyridine group in **2**. The positioning of this atom is near a cleft between the pyridine ligands, which could be a potential site of O_2_ binding and reduction. Consequently, complex **2** has the potential for a possible proton relay in the second coordination sphere that is lacking in **1**. Such second-sphere proton relays have previously been shown to be very effective in enhancing the efficacy of the oxygen reduction reaction [8,25,27,28].

**Figure 2:**
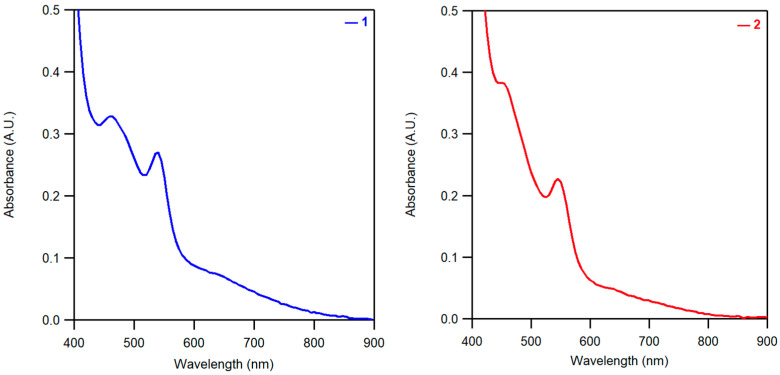
Electronic absorption spectra of 2.0 mM solutions of **1** (**left**) and **2** (**right**) in MeCN at 25 °C.

The electronic absorption spectra of **1** and **2** dissolved in MeCN are quite similar (Figure 2). Each spectrum shows a broad band with a low intensity near ~650–700 nm, a sharper, more intense feature near ~520–550 nm, and a broader, higher-intensity band at ~450 nm. Specifically, complex **1** exhibits electronic absorption bands at 650, 540, and 460 nm (ε = 50, 180, and 150 M^−1^ cm^−1^, respectively). Complex **2** has electronic absorption bands at 650, 545, and 450 nm (ε = 20, 115, and 190 M^−1^ cm^−1^, respectively). In each case, these bands arise from ligand-field transitions of the Co^II^ centers, which are quite sensitive to the metal coordination geometry [29]. Assuming an idealized C_3v_ point group for **1** and **2**, we can assign the three absorption bands as excitations from the ^4^A_2_ ground state to ^4^E, ^4^A_2_(P), and ^4^E(P) excited states, in ascending order. These assignments follow those of Lever, who reported that high-spin, trigonal bipyramidal Co^II^ complexes have three ligand-field absorption bands in the visible spectral region, with ^4^A_2_ → ^4^E at 990–670 nm, ^4^A_2_ → ^4^A_2_(P) at 750–580 nm, and ^4^A_2_ → ^4^E(P) at 580–470 nm [29]. The similarity in the energies and intensities of the electronic absorption bands is consistent with **1** and **2** having the same Co^II^ geometry in MeCN solution. We also performed experiments to determine if MeCN coordinates to the Co^II^ centers in **1** and **2**, which would generate six-coordinate species. Because the electronic absorption spectra of **1** and **2** in the non-coordinating solvent CH_2_Cl_2_ have band energies identical to those in MeCN (Supplementary Materials, Figure S2), we conclude that MeCN does not coordinate to the Co^II^ centers and they remain five-coordinate.

Figure 1 and Figure 2 were obtained from ESI-MS and ^1^H NMR experiments. The ESI-MS data for **1** and **2** at 25 °C show prominent peaks at *m*/*z* of 455.12 and 456.11, respectively (SI Figure S2). These results are in excellent agreement with the calculated *m*/*z* of [Co^II^(PaPy_2_Q)]^+^ (455.12) and [Co^II^(PaPy_2_N)]^+^ (456.11). The ^1^H NMR spectra of **1** and **2** in MeCN-d_3_ are quite similar (Table 1 and Figure S3), with each showing nine peaks in the downfield region (150–10 ppm) and one peak in the upfield region (~−6 to −10 ppm). This large range of chemical shift values is attributed to hyperfine interactions between the protons of the PaPy_2_Q and PaPy_2_N supporting ligands and the paramagnetic Co^II^ centers. Although the development of detailed assignments for these peaks is beyond the scope of this work, the similar chemical shift patterns and identical number of hyperfine-shifted peaks suggest similar structures for **1** and **2** in solution. On the basis of the X-ray crystal structures for **1** and **2** showing equivalent pyridyl groups, we would predict 13 proton resonances for **1** and 12 for **2**. The appearance of fewer than the predicted number of resonances is not unexpected. Protons closest to the Co^II^ center could be fast-relaxing and too broad to observe. Alternatively, some proton resonances might appear in the diamagnetic region and be obscured by solvent peaks.

The perpendicular-mode X-band EPR spectra of frozen solutions of **1** and **2** in MeCN at 7.5 K show positive signals at g ≈ 4.4 and broad, negative signals at higher fields (Table 1 and Supplementary Materials, Figure S4). These signals are similar to those observed for other high-spin Co^II^ complexes [30] and indicate an S = 3/2 ground state. We further probed the spin states of **1** and **2** by performing Evans NMR experiments in MeCN-d_3_ at 25 °C (Table 1 and Figure S5). From these experiments, we observed magnetic moments of 2.6 μB for **1** and 2.9 μB for **2**. (In calculating the magnetic moments for **1** and **2**, we did not include diamagnetic corrections, which are usually small for these types of complexes.) These values are lower than expected for the spin-only magnetic moments of an S = 3/2 system (~3.9 μB) [31]. It is possible that the magnetic moments for **1** and **2** were lower than expected due to reactions with dioxygen that would give product(s) with lower spin states. However, larger ranges of magnetic moments of 2.3–4.3 μB have been reported for Co^II^ complexes [32], which have been attributed to a range of factors [33].

### 2.2. Reduction of Dioxygen with ***1*** and ***2***

#### 2.2.1. Chemical Reduction of Dioxygen

**Figure 3:**
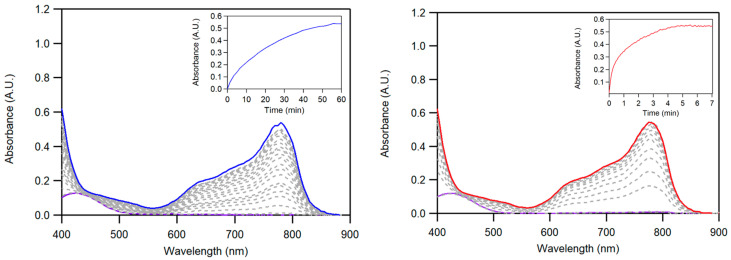
UV–Vis spectrum of the formation of Me_10_Fc^+^ in the presence of a catalytic amount (0.02 mM) of **1** (**left**) and **2** (**right**) in MeCN under air. The initial spectra are in purple, intermediate spectra are gray, dashed traces, and final spectra are blue (**left**) and red (**right**). Conditions: 1.1 mM Me_10_Fc and 15 mM TFA at 25 °C. Insets: Time trace of the formation of Me_10_Fc^+^ at 780 nm.

We explored the ability of **1** and **2** to catalytically reduce dioxygen in an air-saturated MeCN solution at 25 °C using a chemical reductant. We chose decamethylferrocene (Me_10_Fc) as the reductant, with trifluoroacetic acid (TFA) as acid. The reactions used a large excess of reductant (1.1 mM) and acid (15 mM) compared to the cobalt catalyst (0.02 mM). The catalytic ORR activity was determined by monitoring the oxidation of Me_10_Fc to Me_10_Fc^+^ at 780 nm (ε = 495 M^−1^ cm^−1^) by UV–Vis spectroscopy (Figure 3) [34].

The addition of 0.02 mM of **2** to a solution of Me_10_Fc (1.1 mM) and TFA (15 mM) under air led to the complete (100%) conversion of Me_10_Fc to Me_10_Fc^+^ in 5.2 min (Figure 3, bottom). Complex **1** also reacted with 1.1 mM Me_10_Fc and 15 mM TFA, but in this case, the complete conversion of Me_10_Fc to Me_10_Fc^+^ was not observed until 60 min (Figure 3, top). Control reactions in the absence of complexes **1** and **2** gave the maximum formation of Me_10_Fc^+^ at 90 min (Supplementary Materials, Figure S6). The difference in Me_10_Fc^+^ formation rates between **1** and **2** is attributed to the presence of the naphthyridine group in **2** that can engage in second-sphere hydrogen bonding interactions. To provide a detailed comparison of the performances of **1** and **2**, we calculated TOFi (initial turn over frequency) values for each catalyst with respect to the concentration of Me_10_Fc^+^ (Supplementary Materials, Figure S7). On the basis of these values, complex **2** (TOFi = 1200 h^−1^) reacted ~23 times more rapidly than complex **1** (TOFi = 70 h^−1^).

To assess the robustness of **1** and **2** as catalysts, we performed cycles of catalytic experiments using 0.02 mM Co complex, 15 mM TFA, and 0.55 mM (0.36 mmoles) Me_10_Fc. After Me_10_Fc^+^ had reached maximal formation, 0.36 mmoles of Me_10_Fc was added to the same reaction mixture. Each addition resulted in the complete conversion of the added Me_10_Fc to Me_10_Fc^+^ (Supplementary Materials, Figure S8). An increase in the reaction time was observed for the second and third Me_10_Fc additions. Specifically, the reaction time for **1** increased by ~3 min for each addition of Me_10_Fc. For **2**, the reaction time increased by ~5 min. These observations imply that some fraction of the catalyst might have decayed. However, the reaction times are still less than those observed in the absence of a catalyst (120 min when using 15 mM TFA and 0.55 mM Me_10_Fc), implying that a significant portion of the catalyst remained active over multiple cycles.

The Ti-TPyP (oxo [5,10,15,20-tetra(4-pyridyl)porphyrinato]titanium(IV)) reagent was utilized to quantify the amount of hydrogen peroxide produced from the reduction of dioxygen in the reactions of **1** and **2**. From these experiments, we observed 18 ± 6% formation of H_2_O_2_ for **1** and 27 ± 7% H_2_O_2_ formation for **2**. The percentages are based on the theoretical 100% yield of H_2_O_2_ based on the concentration of 1.1 mM Me_10_Fc (Equation (3)), as follows:

O_2_ + 2 Me_10_Fc + 2 CF_3_CH_2_COOH → 2 Me_10_Fc^+^ + H_2_O_2_ + 2 CF_3_CH_2_COO^−^ (3)

Because hydrogen peroxide is known to react with certain ferrocene derivatives, including Me_10_Fc [35,36,37], the values of H_2_O_2_ reported here should be viewed as lower limits of the actual amount of H_2_O_2_ produced in these reactions. In support, we repeated the catalytic reduction of O_2_ in the presence of **2** at −40 °C while keeping all other conditions the same as those in our experiments at 25 °C (Supplementary Materials, Figure S9). The lower temperature was chosen to minimize the decomposition of H_2_O_2_. We observed 42 ± 5% H_2_O_2_ under these conditions. This result suggests that catalysts **1** and **2** predominantly generate hydrogen peroxide, which decays in the presence of Me_10_Fc at higher temperatures. To further verify this possibility, we repeated the catalytic reactions with H_2_O_2_ (1.1 mM) instead of dioxygen. In the first experiment (Supplementary Materials, Figure S10), H_2_O_2_ (1.1 mM) was added to an MeCN solution of Me_10_Fc (1.1 mM), TFA (15 mM), and complex **2** (0.02 mM) under a nitrogen atmosphere at 25 °C. After approximately 30 s, we observed ~100% conversion of Me_10_Fc to Me_10_Fc^+^ and detected 52 ± 3% of hydrogen peroxide in the final solution. This near 50% conversion of H_2_O_2_ is consistent with two equiv. of Me_10_Fc consumed per one equiv. of H_2_O_2_ consumed (i.e., Me_10_Fc is a one-electron reductant, and the reduction of H_2_O_2_ to H_2_O requires two electrons). A second experiment performed in the absence of a catalyst (Supplementary Materials, Figure S11) revealed ~100% formation of Me_10_Fc^+^ in roughly 30 s, with ~56 ± 9% of hydrogen peroxide remaining. These data suggest that H_2_O_2_ is rapidly reduced to H_2_O by Me_10_Fc. On this basis, we conclude that H_2_O_2_ is the dominant product of dioxygen reduction by **1** and **2** under the described conditions.

#### 2.2.2. Electrocatalytic ORR by Complexes **1** and **2**

**Figure 4:**
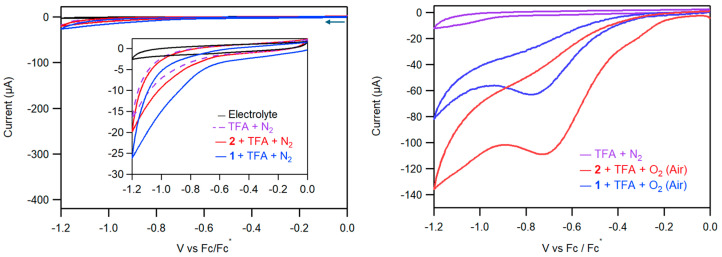
(**Left**): CVs of 0.2 mM solutions of complexes 1 (red) and 2 (blue) in MeCN, under nitrogen, in the presence of TFA (5 mM). Inset: Zoomed in cyclic voltammograms under nitrogen. (**Right**): Cyclic voltammograms of 0.1 mM solutions of the complexes 1 (red trace) and 2 (blue trace) in MeCN saturated with oxygen, in the presence of TFA (5 mM). All CVs are conducted using a 3 mm glassy carbon working electrode and a Pt wire counter electrode at a scan rate of 0.1 V/s.

The abilities of **1** and **2** to electrocatalytically reduce dioxygen were investigated with cyclic voltammetry (CV). These experiments were performed in an MeCN solution of 0.1 M tetrabutylammonium hexafluorophosphate (TBAPF_6_) at room temperature. A background scan of the electrolyte solution shows no current with a magnitude greater than -3 mA in the potential range from 0 to −1.2 V vs. Fc/Fc+ (Figure 4, left inset). A scan with only electrolyte and TFA (5 mM) under a nitrogen atmosphere shows a slight increase in current magnitude to approximately −15 μA near 1.2 V (Figure 4, left, and Supplementary Materials, Figure S12). The magnitude of this current increased only slightly for solutions of electrolyte, TFA, and a catalytic amount (0.2 mM) of either **1** or **2** under nitrogen (Figure 4, left). Large changes are observed in the CV traces when the same experiments with 0.1 mM solutions of **1** and **2** were performed in an oxygen-saturated solution. In the presence of a catalytic amount of **2** (0.1 mM) (Figure 4, right, red trace), an irreversible peak is observed at −0.71 V vs. Fc^+^/Fc, exhibiting a large catalytic current (more than −100 μA). Similarly, a CV scan for a solution of electrolyte, TFA, and **1** (0.1 mM) in the presence of oxygen shows an irreversible peak at −0.77 V vs. Fc^+^/Fc with a current near −60 μA (Figure 4, right, blue trace). Notably, the catalytic current of **2** was ~1.6-fold larger than that of **1** (Figure 4, left). This difference in the magnitude of the produced catalytic current is potentially due to the difference in the second coordination spheres of these two complexes (Figure 1).

**Figure 5:**
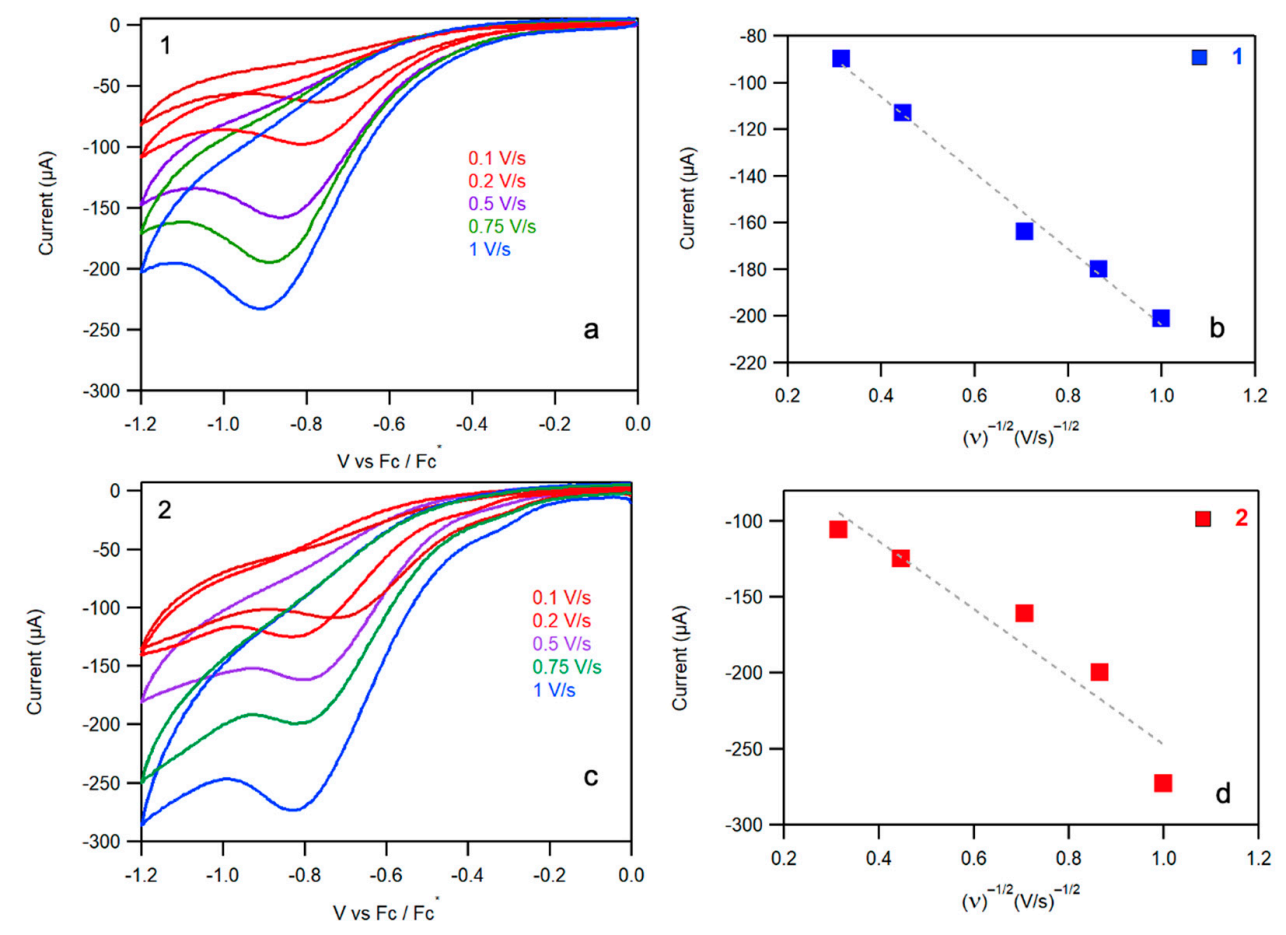
Top left (**a**): CV of 0.1 mM complex **1**, in a solution of 5 mM TFA in MeCN saturated with oxygen at different scan rates. Down left (**c**): CV of 0.1 mM complex **2**, saturated with oxygen in a solution of 5 mM TFA in MeCN at different scan rates. Right (**b**,**d**): A plot of catalytic current vs. square root of scan rate of complex **1** (**b**) and **2** (**d**).

The scan rate dependence of the catalytic current was evaluated by CV experiments in an oxygen-saturated solution in the presence of 5 mM TFA at room temperature. Both cobalt complexes showed a linear relationship between the square root of the scan rate vs. peak current (Figure 5), indicating that the electrocatalytic process was diffusion-controlled [8,38,39]. This result suggests that **1** and **2** perform electron transfer to reduce O_2_ as homogeneous species and not as adsorbed catalysts on the electrode surface. If adsorption was occurring, the peak current would be expected to show a linear relationship with the scan rate [39].

## 3. Discussion

The controlled reduction of dioxygen is critical to both biological and industrial processes. In a key biological example, dioxygen serves as a proton and electron acceptor during cellular respiration in aerobic organisms. In this multi-step process, the reduction of O_2_ to H_2_O is coupled with proton transfer across a membrane, driving ATP synthesis [40]. Emerging energy technologies such as fuel cells and metal–air batteries utilize the reduction of O_2_ to H_2_O to convert chemical to electrical energy [2]. In both systems, catalysts are required to coordinate the delivery of multiple protons and electrons to O_2_. At present, the most effective synthetic catalysts are carbon-supported platinum nanoparticles [2]. The high cost and low abundance of platinum causes scalability issues with fuel cells, motivating the development of new catalysts with earth-abundant metals [13,41,42,43,44]. To develop these new catalysts, we must understand the specific structural and electronic factors that control (a) the efficiency of O_2_ reduction and (b) the selectivity for forming H_2_O. Structure–function performance metrics are most readily obtained for homogeneous catalysts. The knowledge gained from these studies can be applied to optimize heterogeneous catalysts, which are more practical in fuel cells. While certain catalyst design elements, such as potential sites of protonation (Lewis bases) near the O_2_ binding site, have been shown to affect both O_2_ reduction efficiency and selectivity, more systematic studies are needed to link particular molecular features with increases in performance.

**Scheme 3:**
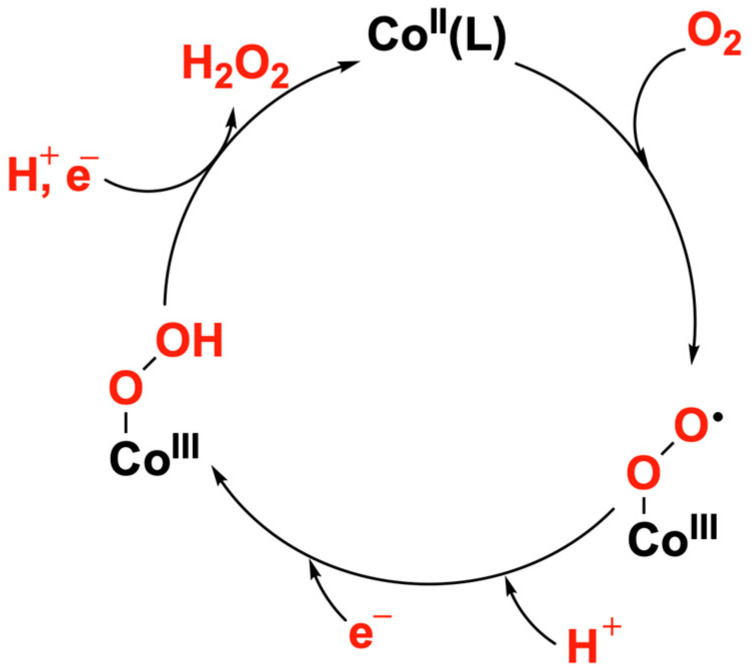
General proposed mechanism for ORR by mononuclear Co complexes.

In this present study, we generated a pair of cobalt complexes (**1** and **2**; see Figure 1), which allowed us to evaluate the role of a Lewis base in close proximity to the metal center in oxygen reduction selectivity and efficiency. Both X-ray crystallography and spectroscopic measurements revealed that **1** and **2** had very similar Co^II^ coordination environments. However, the naphthyridine moiety in **2** incorporated a Lewis base in the second coordination sphere. Several previous studies have established that second-sphere Lewis bases can enhance the rate of dioxygen reduction and can also modulate the selectivity for H_2_O versus H_2_O_2_ production [8,25,28,29]. These second-sphere bases likely assist in the delivery of protons to Co–oxygen intermediates. Scheme 3 shows a general mechanism for oxygen reduction by a mononuclear Co catalyst [24]. Oxygen reduction is initiated by the binding of O_2_ to the Co^II^ complex to give a Co^III^-superoxide (Co^III^-OO.) intermediate. This intermediate is protonated and reduced (either in a concerted or stepwise fashion), generating a Co^III^-hydroperoxo (Co^III^-OOH) intermediate. In the last step, the second protonation and reduction occur and are followed by the cleavage of the Co-O bond to hydrogen peroxide, regenerating the Co^II^ species.

In our experiments probing the ability of **1** and **2** to reduce oxygen using the reductant Me_10_Fc, we observed that **2** achieved the maximum formation of Me_10_Fc^+^ 10-fold faster than **1**. Given that the only difference between these complexes was the presence of the second-sphere Lewis base in **2**, we attribute this difference to the role of this Lewis base in facilitating the reduction of dioxygen. We also studied the ability of both complexes to reduce dioxygen electrochemically. CV measurements showed that **2** had a ~1.6-fold larger catalytic current compared to **1**. Although the Lewis base in **2** had a large influence on the rate of dioxygen reduction, the effect on product selectivity appeared more limited. An analysis of the reaction mixtures following the chemical reduction of dioxygen at 25 °C revealed 20 ± 6% and 30 ± 7% formation of H_2_O_2_ for **1** and **2**, respectively. In both cases, this extent of H_2_O_2_ formation should be viewed as a lower limit, given the potential for Me_10_Fc to facilitate the reduction of H_2_O_2_ to H_2_O. The reduction of dioxygen to H_2_O_2_ is well-known for cobalt ORR catalysts [24,44,45,46,47,48,49,50], although shifts from all N-coordination to mixed N/O or N/S coordination appear to increase the selectivity for H_2_O [11,24].

What makes complex **2** a more efficient catalyst than **1**? In a recent study of a different mononuclear Co ORR catalyst, Machan and co-workers demonstrated that the proton-transfer steps to the Co–oxygen intermediates were rate-determining [24]. Thus, any modifications to the catalyst architecture that would facilitate these steps could increase catalytic performance. We postulate that, in the presence of an acid such as TFA, complex **2** exists as an equilibrium of protonated forms, with N6 (Figure 1) as the site of protonation. The ability of **2** to harbor protons near the Co center could enhance the rate of proton-transfer steps, thereby increasing its catalytic efficiency for dioxygen reduction. Alternatively, or in addition, the ability of **1** to perform dioxygen reduction could be suppressed by the relatively close proximity of H24 (Supplementary Materials, Figure S1) to the Co center. This hydrogen is 3 Å from the Co center and, therefore, could slightly disfavor dioxygen coordination relative to **2**.

While the comparison of **1** and **2** provides a convenient probe for the influence of second-sphere effects on the reduction of dioxygen, the overall catalytic performance of these complexes is not as high as that of other homogeneous complexes. For example, a recently reported thiolate-ligated dicobalt catalyst had a TOFi = 3000 h^−1^ for the reduction of O_2_ to water, using Me_8_Fc as a reductant and LutHBF_4_ as the proton source [11]. This TOFi is significantly faster than those of **1** and **2** (TOFi = 70 and 1200 h^−1^, respectively). Thus, catalysts that incorporate advantageous features of the primary and secondary coordination spheres of these systems might be best suited for achieving a high performance.

## 4. Materials and Methods

### 4.1. General Methods and Instrumentation

All chemicals were utilized as purchased from commercial suppliers unless stated otherwise. The PaPy_2_QH (N,N-Bis(2-pyridylmethyl)amine-N-ethyl-2-quinolinecarboxamide) and PaPy_2_NH (N-(2-(bis(pyridine-2-ylmethyl)amino)ethyl)-1,8-naphthyridine-2-carboxamide) ligands were synthesized by previously reported methods [26,51].

Electronic absorption measurements were acquired by an Agilent 8453 UV–Vis system (Agilent, Santa Clara, CA, USA) or a Varian Cary 50 Bio UV–Vis spectrophotometer (Varian, Cranford, NJ, USA). The reaction temperature was regulated using a Quantum Northwest TC 1 temperature controller and stirrer (Quantum Northwest, Liberty Lake, WA, USA) or a Unisoku cryostat and stirrer (Unisoku, Hirakata, Japan). ^1^H NMR measurements were collected with a Bruker DRX 400 MHz spectrometer (Bruker, Billerica, MA, USA). The hyperfine-shifted ^1^H NMR spectra were obtained over an expanded spectral window (from 150 to −100 ppm) using 1000 scans to enhance S/N. Spline-based baseline correction was obtained using multipoint spline adjustment in the MestReNova software (version 16.0.0-39276). ESI-MS (electrospray ionization mass spectrometry) measurements were carried out on an LCT Premier Micromass electrospray time-of-flight mass spectrometer (Micromass, Cary, NC, USA). The X-band EPR analysis was performed using a Bruker EMXplus spectrometer (Bruker, Billerica, MA, USA) equipped with an Oxford ESR900 continuous-flow liquid helium cryostat and controlled via an Oxford ITC503 temperature unit (Oxford Instruments, Concord, MA, USA).

### 4.2. Synthesis of ***1*** and ***2***

#### 4.2.1. Synthesis of [Co^II^(PaPy_2_Q)](OTf) (**1**)

In a glovebox, 0.351 g (0.88 mmol) of PaPy_2_QH was dissolved in 20 mL of CH_3_OH. A solution of 0.085 g (0.88 mmol) of NaOtBu dissolved in 20 mL of CH_3_OH was added dropwise to the solution of PaPy_2_QH under vigorous stirring. The mixture was stirred continuously for 15 min. Then, 0.316 g (0.88 mmol) of Co^II^(OTf)_2_ in 20 mL of CH_3_OH was added dropwise to the mixture. A cloudy brown solution was obtained. The mixture was stirred continuously for 24 h. The reaction was stopped, and the resulting brown solution was filtered using a syringe filter. The solvent was removed completely from the filtrate, leaving behind a reddish-brown precipitate. The solid was dissolved in approximately 10 mL of MeCN and layered with ether for recrystallization. After the ether layer and MeCN layer diffused into each other to form a single layer with precipitated solids, the solvent was carefully decanted, leaving behind some reddish-brown solid. The precipitate was dried in vacuo, washed with ether, and repeatedly dried two times. Repeated recrystallization of this reddish-brown solid in MeCN produced 0.462 g of **1** (87% yield). Crystals suitable for X-ray crystallography were obtained by the slow vapor diffusion of ether into a concentrated acetonitrile solution of **1**.

#### 4.2.2. Synthesis of Na[Co^II^(PaPy_2_N)](OTf)_2_ (**2**)

In a glovebox, 0.429 g (1.1 mmol) of PaPy_2_NH was dissolved in 25 mL of CH_3_OH. A solution of 0.104 g (1.1 mmol) of NaOtBu dissolved in 20 mL of CH_3_OH was added dropwise to the solution of PaPy_2_NH under vigorous stirring. The mixture was stirred continuously for 15 min. Then, 0.385 g (1.1 mmol) of Co^II^(OTf)_2_ in 20 mL of CH_3_OH was added dropwise to the mixture. A pink-brown solution was obtained. The mixture was stirred continuously for 24 h. The reaction was stopped, and the resulting brown solution was filtered using a syringe filter. The solvent was removed completely from the filtrate, leaving behind a reddish-brown precipitate. The solid was dissolved in approximately 15 mL of MeCN and layered with ether for recrystallization. After the ether layer and MeCN diffused into each other to form a single layer with precipitated solids, the solvent was carefully decanted, leaving a reddish-brown solid. The solid was dried in vacuo, washed with ether, and repeatedly dried two times. Repeated recrystallization of this reddish-brown precipitate in MeCN produced 0.524 g of **2** (80% yield). Crystals suitable for X-ray crystallography were obtained by the slow vapor diffusion of ether into a concentrated acetonitrile solution of **2**.

### 4.3. XRD Characterizations

Complete sets of unique reflections were collected with monochromated CuKα radiation for single-domain crystals of both compounds. Totals of 2619 ([Co^II^(PaPy_2_Q)][OTf]) and 3931 (Na[Co^II^(PaPy_2_N)][OTf]_2_) 1–wide ω- or ϕ-scan frames were collected with a Bruker APEX II CCD (Bruker, Billerica, MA, USA)area detector. Frame-counting times of 5–8 s were used for [Co^II^(PaPy_2_Q)][OTf] and 4–6 s for Na[Co^II^(PaPy_2_N)][OTf]_2_. X-rays for both structures were provided by a Bruker MicroStar (Bruker, Billerica, MA, USA)microfocus rotating anode operating at 45 kV and 60 mA and equipped with an APEX II CCD (Bruker, Billerica, MA, USA)detector and Helios multilayer X-ray optics. Preliminary lattice constants were obtained with the Bruker program SMART. Integrated reflection intensities for both crystals were produced using the Bruker program SAINT [52]. Both data sets were corrected empirically for variable absorption effects using SADABS [52]. SHELXT [53] was used to solve each structure using intrinsic phasing/dual-space techniques. All stages of weighted full-matrix least-squares refinement were conducted using Fo^2^ data with SHELXL v2017 [54].

The final structural model for both structures incorporated anisotropic thermal parameters for all non-hydrogen atoms and isotropic thermal parameters for all hydrogen atoms. In order to experimentally distinguish between the PaPy_2_Q and PaPy_2_N ligands, the hydrogen atom bonded to the C24 atom in the PaPy_2_Q ligand of **1** was located from a difference Fourier and included in the structural model as an independent isotropic atom whose parameters were allowed to vary in least-squares refinement cycles. The C24–H24 bond length in **1** was refined to a final value of 0.86(5) Å. No significant electron density was observed at a reasonable sp^2^-hybridized position around the corresponding nitrogen atom in the PaPy_2_N ligand of **2**. The remaining ligand hydrogen atoms in both structures (except H24 in **1**) were placed at idealized riding model sp^2^- or sp^3^-hybridized positions with C–H bond lengths of 0.95–0.99 Å. The isotropic thermal parameters of the idealized hydrogen atoms in both structures were fixed at values 1.2 times the equivalent isotropic thermal parameter of the carbon atom to which they are covalently bonded. The relevant crystallographic and structure refinement data from these models for both compounds are given in Tables S1 and S2. Due to physical limitations of the instrument with Cu radiation, it was difficult to obtain triclinic and monoclinic Cu data sets with 100% completeness without remounting the data crystal. Both data sets, therefore, had more than a hundred reflections each that were not measured.

An alternate refinement model for **1** was proposed by a reviewer who noted that “SHELXL uses spherical atomic factors (so called ‘IAM model’) for all atoms. This results in significant bias in hydrogen atom location; among other things: C–H bonds are shorter by 0.1–0.2 A. Therefore, refinement of H atom locations using X-ray data with this model results in a good fit but not in the correct position of hydrogen atoms. The simplest correction for achieving correct hydrogen positions is to assign a (neutron diffraction) value of 1.083 Å to all C–H bonds. Alternatively, non-spherical factors from DFT calculations for each specific structure can be calculated and used for refinement. The resulting bias is less than 0.02 A (which is within the usual standard deviation for X-H of 0.02–0.04 A).” The reviewer ran *olex-refine* with *NoSpherA2* (*ORCA5.0*, *r2SCAN*, *BASIS SET: def2-TZVP*, *MULTIPLICITY: 4*). The final C24–H24 bond length from this refinement was 1.05(5) Å, a more realistic value than 0.86(5) Å.

Of course, the most important point is that all reasonable models should, and do, in this case, indicate the unequivocal presence of H24 as part of a C–H moiety in **1** and its absence in **2**, where the C–H group is replaced by an isoelectronic and chemically useful N atom.

### 4.4. Catalytic Reduction of Dioxygen

#### 4.4.1. Chemical Catalytic ORR by UV–Vis Spectroscopy

Measurements of UV–Vis spectroscopy were performed in a quartz cuvette with a 1 cm pathlength at 25 °C under air. In total, 300 μL of a 7 mM stock solution of Me_10_Fc was introduced to 1635 μL of acetonitrile (MeCN) in the cuvette, followed by the addition of 25 μL of TFA from a 1.2 M stock solution. After approximately five seconds, to allow for the mixing of TFA and Me_10_Fc, 40 μL of the catalyst solution (1 mM of stock solution in MeCN) was added to the mixture. In the control experiments, 40 μL of MeCN was added to the mixture instead of the catalyst solution.

#### 4.4.2. Electrocatalytic ORR by CV

Electrochemical measurements were conducted using a three-electrode system equipped with a glassy carbon working electrode, a platinum wire counter electrode, and a non-aqueous reference electrode (containing supporting electrolyte solution). All solutions were prepared in 0.1 M TBAPF_6_ in MeCN solution. For each experiment, 50 μL of a 0.3 M TFA stock solution was added to the cell containing 2.8 mL of the supporting electrolyte solution, followed by the addition of 150 μL of a 5 mM catalyst stock solution prepared under an inert atmosphere. This mixture was saturated with oxygen by passing oxygen gas through the solution. In order to evaluate the activity of the catalyst in the absence of oxygen, experiments were carried out with identical conditions under a nitrogen atmosphere.

### 4.5. Quantification of Produced H_2_O_2_ from Chemical Catalytic ORR

To determine the amount of H_2_O_2_ generated in each catalytic dioxygen reduction reaction, spectroscopic titrations were performed with an acidic solution of [TiO(TPyPH_4_)]^4+^ (Ti-TPyP reagent) [55,56]. The reagent solution was prepared by dissolving [TiO(TPyP)] (≥90%, TCI) (3.44 mg) in 100 mL of 0.05 M aqueous HCl. This solution is light-sensitive and was stored at 5 °C in a refrigerator.

A small portion (15 μL) of each sample solution (collected 10 min after reaction completion) was added to a mixture of the Ti-TPyP reagent (250 μL), 4.8 M perchloric acid (250 μL), and water (235 μL) at 20 °C. After 5 min, the resulting solution was diluted to a final volume of 2.5 mL with water, and the absorbance at λ = 433 nm was measured in a 1 cm pathlength UV–Vis cuvette. In a similar manner, a blank solution was prepared by adding 15 μL of the solvent (acetonitrile) instead of the sample solution. All experiments were repeated three times to obtain reproducible data in the 2% range. A decrease in the sample absorbance is indicative of the presence of H_2_O_2_ in the solution. (ΔA_sample(i)_ = A_blank_ − A_sample(i)_). To perform the calibration, the same procedure was conducted by adding 15 μL of aqueous solutions of H_2_O_2_ (in the range from 100 to 500 μL, prepared via the dilution of a standard solution of 9.66 mM) instead of sample solutions. By plotting the ΔA values (ΔA_cal(i)_) against the concentration of H_2_O_2_ in the final sample solution ([H_2_O_2_]_final sample_) (Supplementary Materials, Figure S13), Equation (4) is obtained as follows:

∆A = 0.1546 [H_2_O_2_]_final sample_ + 0.042 (4)

The amount of H_2_O_2_ in the final solution ([H_2_O_2_]_final sample_) was determined from A_sample(i)_ via Equation (4).

## Figures and Tables

**Table 1:** Cobalt–ligand bond lengths (Å) from X-ray crystallography, UV–Vis absorption band maxima (nm), and intensities (M^−1^ cm^−1^), ^1^H NMR chemical shifts (ppm), EPR *g* values, and solution magnetic moments (μB), for complexes **1** and **2**.

Property	1	2
	L = PaPy_2_Q	L = PaPy_2_N
Cobalt–ligand bond lengths		
Co–N1	2.107 (3)	2.0806 (19)
Co–N2	1.935 (3)	1.959 (2)
Co–N3	2.182 (3)	2.1914 (19)
Co–N4	2.036 (3)	2.041 (2)
Co–N5	2.058 (3)	2.0503 (19)
λ_max_ (ε)	650 (50)	650 (20)
	540 (180)	545 (115)
	460 (150)	450 (190)
^1^H NMR	149.1	141.5
	132.6	121.2
	79.9	95.7
	77.4	90.4
	65.6	73.0
	55.5	65.9
	53.9	53.8
	44.3	45.8
	−1.5	−0.4
	−7.4	−5.9
*g*	~4.4	~4.4
	~2.0	~1.9
μ_eff_	2.6	2.9

## Data Availability

Crystallographic data are available through the Cambridge Crystallographic Data Centre (CCDC) at https://www.ccdc.cam.ac.uk/ with structure codes 2465463 (complex **1**), and 2465462 (complex **2**).

## Supplementary Materials

The following supporting information can be downloaded at: https://www.mdpi.com/article/10.3390/molecules30153274/s1, Figure S1: X-ray crystal structure of **2**, Figure S2: Top: ESI-MS of **1** and **2**. Bottom: UV–Vis spectra of **1** and **2** in CH_2_Cl_2_, Figure S3: ^1^H NMR of **1** and **2**, Figure S4: Perpendicular-mode EPR data of **1** and **2**, Figure S5: Evans NMR spectra of **1** and **2**, Figure S6: UV–vis spectra of control experiment, Figure S7: linear initial rate fit of time profile of ORR in the presence of **1** and **2**, Figure S8: Time profile of catalytic ORR after multiple addition of Me_10_Fc, Figure S9: UV–Vis spectra of ORR by **2** at −40 °C, Figure S10: UV–Vis spectra of H_2_O_2_ reduction in the presence of **2** at 25 °C under N_2_, Figure S11: UV–Vis spectra of H_2_O_2_ reduction in the absence of catalyst at 25 °C under N_2_, Figure S12: Background cyclic voltammograms of TFA under nitrogen and under oxygen, Figure S13: Plot of ΔA_cal(i)_ vs. [H_2_O_2_]_final sample_, Table S1: Crystal data and structure refinement for [Co(C_24_H_22_N_5_O)][O_3_SCF_3_] (**1**), Table S2: Crystal data and structure refinement for [Na][Co(C_23_H_21_N_6_O)][O_3_SCF_3_]_2_ (**2**).
