# Supplementary material for: Oxygen Reduction by Amide-Ligated Cobalt Complexes: Effect of Hydrogen Bond Acceptor
Source: Molecules. 2025 Aug 5;30(15):3274. doi: 10.3390/molecules30153274 (PMC12348522; doi:10.3390/molecules30153274)
Supplement: Supplementary file 1 [file molecules-30-03274-s001.zip › molecules-3760984-supplementary.pdf]

Supplementary Information for

Oxygen Reduction by Amide-Ligated Cobalt Complexes: Effect of Hydrogen Bond Acceptor

Zahra Aghaei [1], Adedamola A. Opalade [1], Victor W. Day [2] and Timothy A. Jackson [1,*]
1 Department of Chemistry and Center for Environmentally Beneficial Catalysis, University of Kansas,
Lawrence, KS 66045, USA; zahra.aghaei@ku.edu (Z.A.); aaopalade@gmail.com (A.A.O.)
2 X-Ray Crystallography Laboratory, University of Kansas, Lawrence, KS 66045, USA; victorwday@gmail.com
* Correspondence: taj@ku.edu; Tel.: +1-785-864-3968

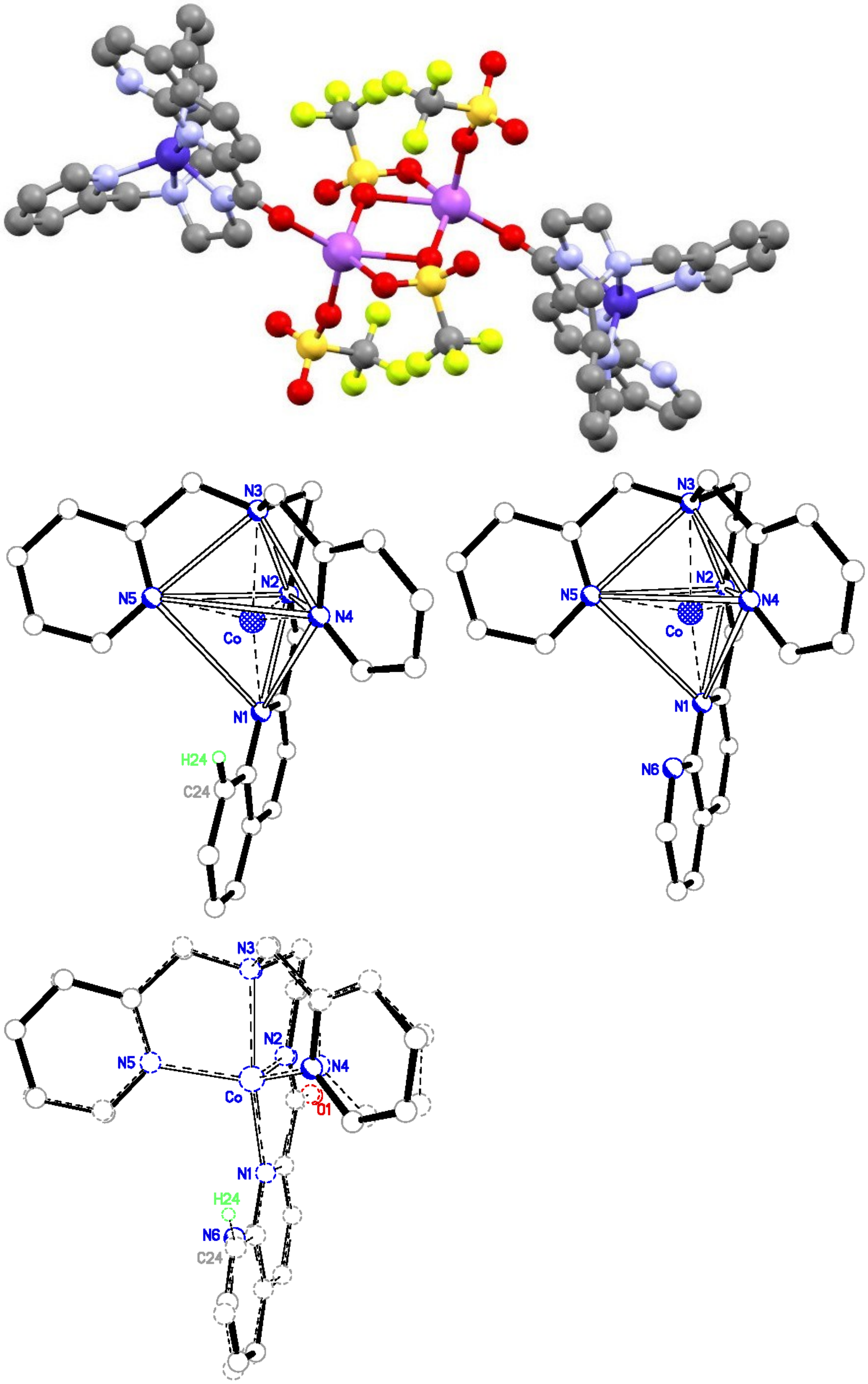

Figure S1. Top: X-ray crystal structure of **2** showing the bridging Na^{+} and triflate ions. Center: Structures of **1** (left) and **2** (right) showing coordination polyhedral. These plots omit all counter ions and the oxygen atoms of the amide function. Bottom: Overlay plot of **1** and **2** with hydrogen atom omitted for clarity.

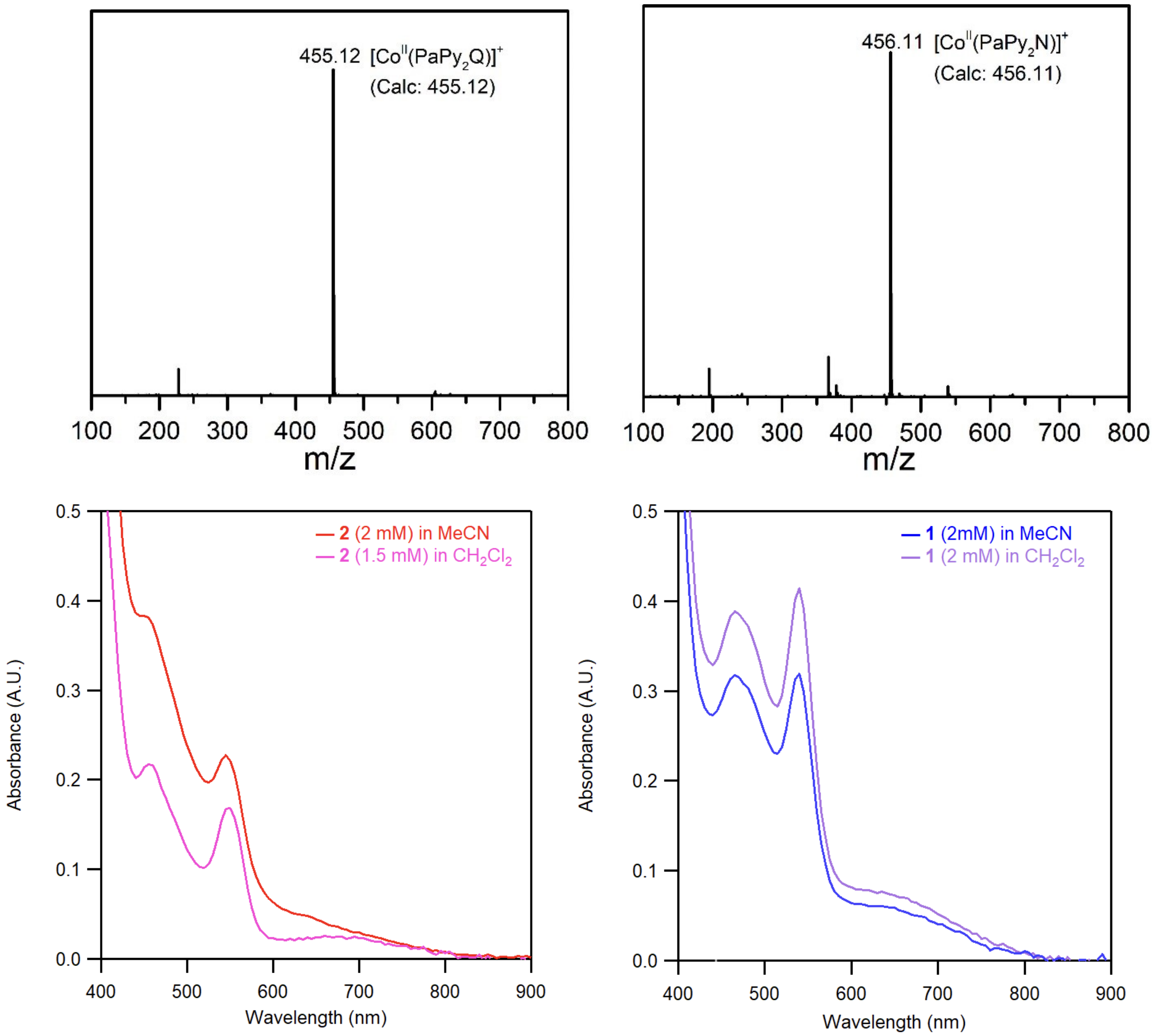

Figure S2. Top: ESI-MS of **1** (left) and **2** (right) in MeCN at 25 °C. Bottom-right: UV-Vis spectra of 2.0 mM solutions of **1** in MeCN (blue trace) and in CH_2Cl_2 (violet trace) at 25 °C. Bottom-left: UV-Vis spectra of 2.0 mM solutions of **2** in MeCN (red trace) and 1.5 mM solutions of **2** in CH_2Cl_2 (pink trace) at 25 °C.

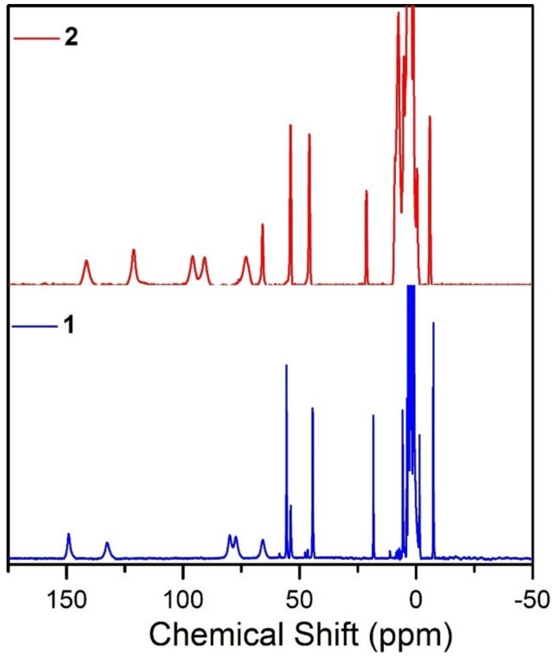

Figure S3. ^{1}H NMR of 30 mM solution of **1** and **2** in MeCN-d_3 at 25 °C.

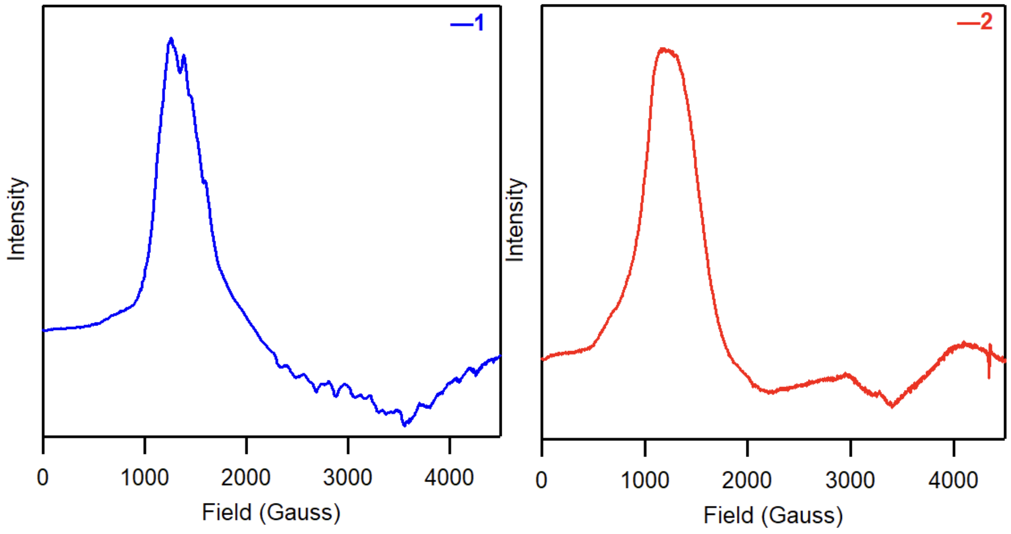

Figure S4. Perpendicular mode, X-band EPR data of 3 mM frozen solutions of **1** (left) and **2** (right) in MeCN at 7.5 K.

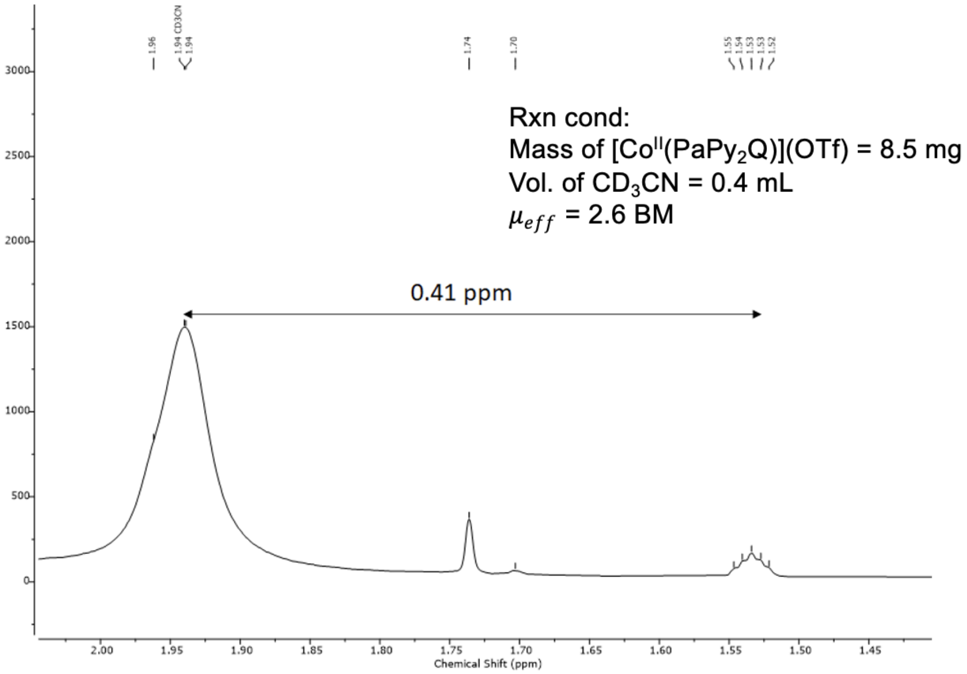

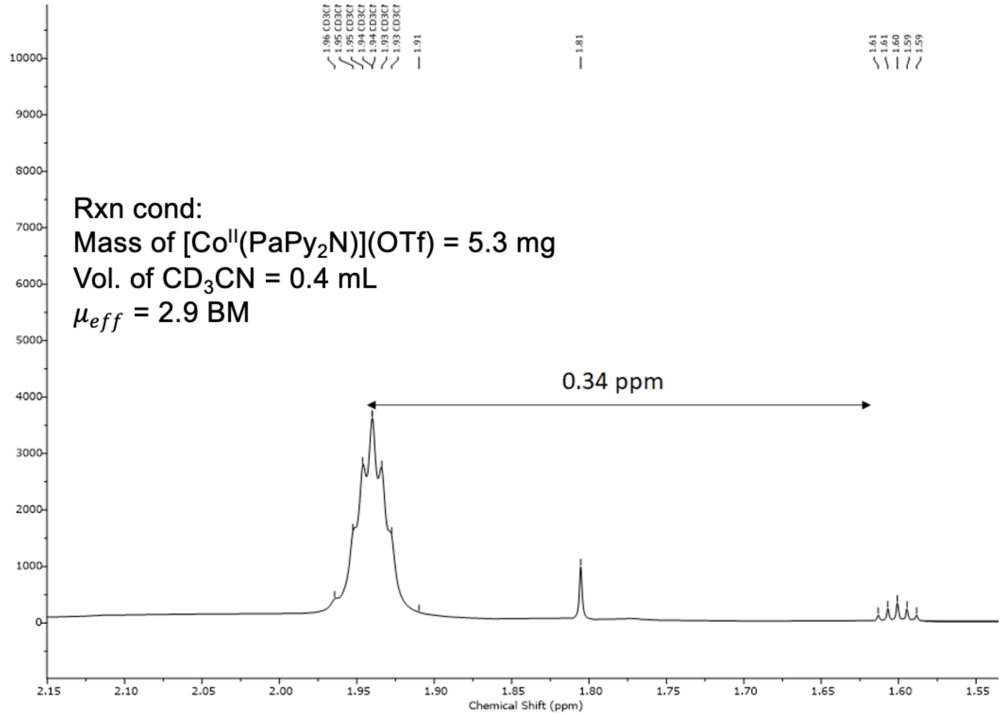

Figure S5. Evans NMR spectra of **1** (top) and **2** (bottom) in MeCN-d_3.

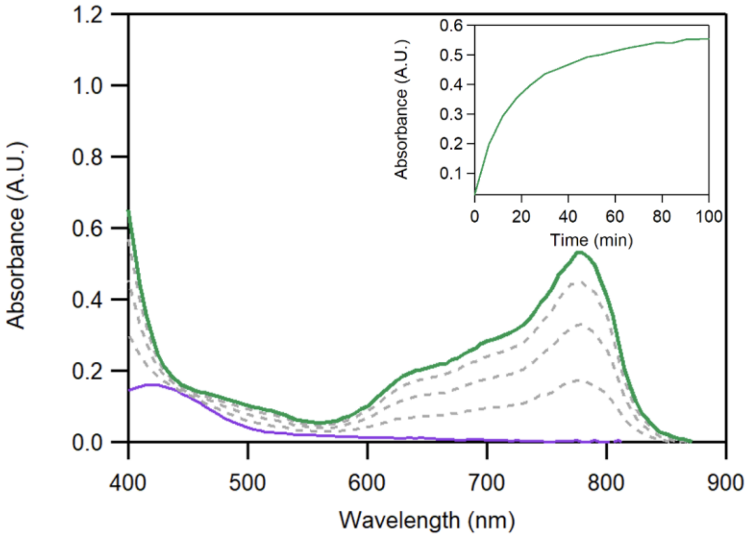

Figure S6. UV-vis spectra of the formation of $Me_{10}Fc^+$ in a MeCN solution of 1.1 mM $Me_{10}Fc$ and 15 mM TFA at 25 °C under air. The inset shows the growth of $Me_{10}Fc^+$ over time.

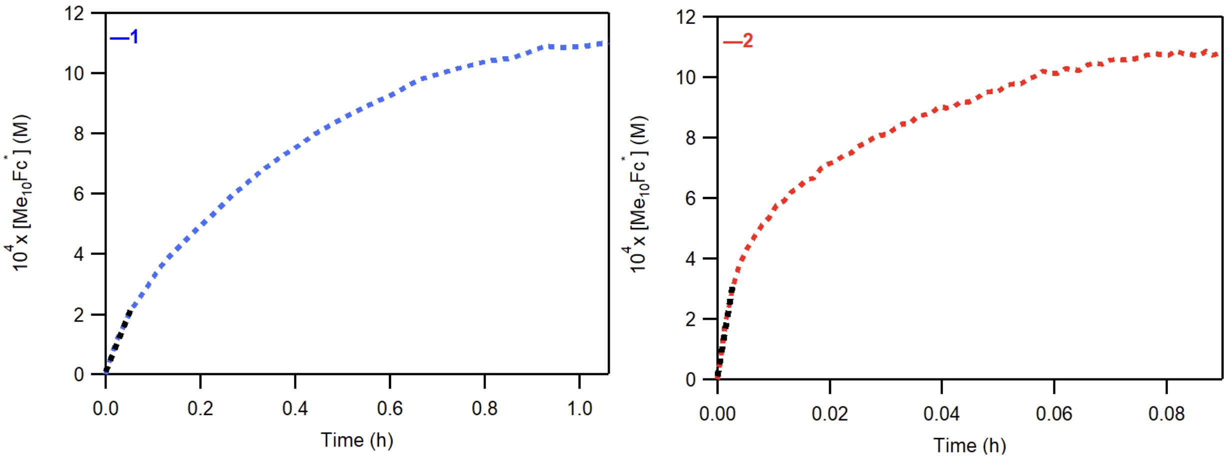

Figure S7. Formation of $Me_{10}Fc^+$ generation at 780 nm in the presence of 0.02 mM of **1** (left) and 0.02 mM of **2** (right). The black dashed line is a linear initial rate fit. Conditions: 1.1 mM $Me_{10}Fc$, 15 mM TFA.

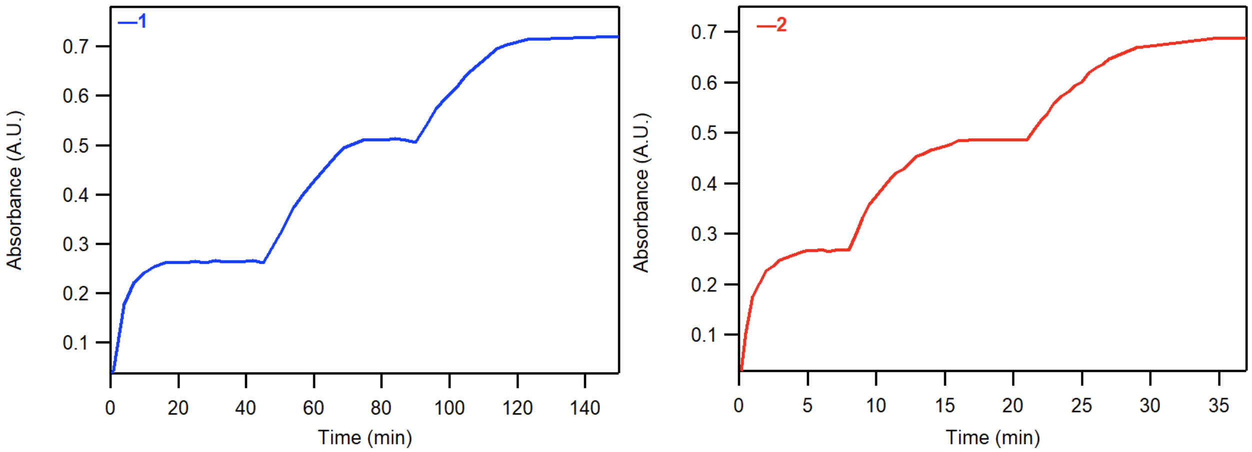

Figure S8. Time profile of $Me_{10}Fc^+$ generation at 780 nm in the presence of 0.02 mM of **1** (left) and 0.02 mM of **2** (right). Conditions: $Me_{10}Fc$ (0.525 mM), TFA (15 mM), in MeCN, under air, at 25 °C.

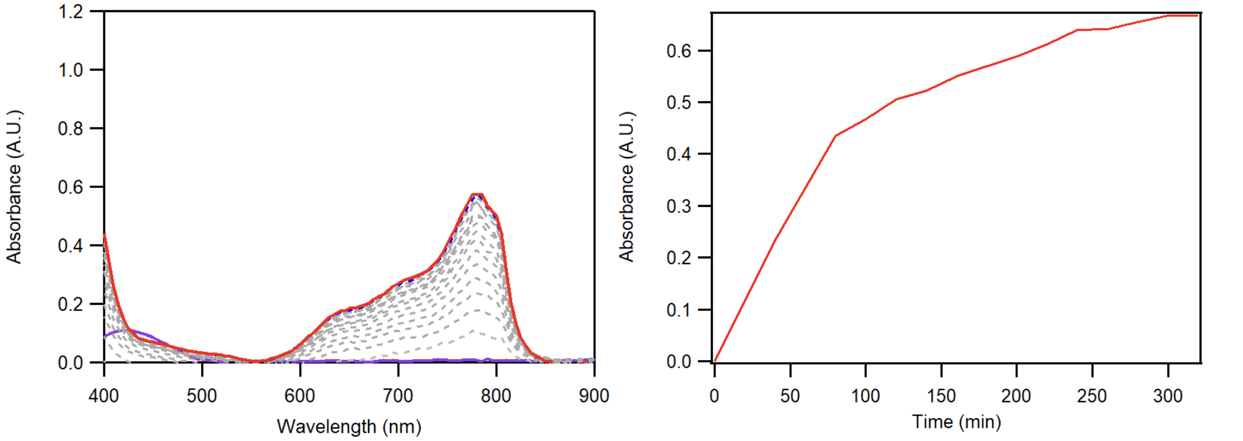

Figure S9. Left: UV−Vis spectra of the formation of $Me_{10}Fc^+$ in a MeCN solution of 0.02 mM **2,** 1.1 mM $Me_{10}Fc$, 15 mM TFA at – 40 °C under air. Right: Time profile (absorbance at 780 nm) for to the formation of $Me_{10}Fc^+$.

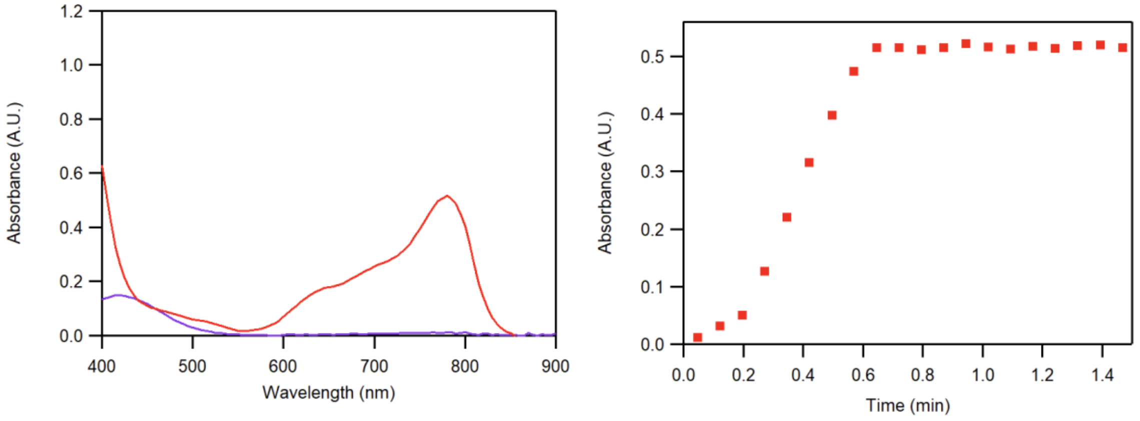

Figure S10. Left: UV−Vis spectra of the formation of $Me_{10}Fc^+$ in a MeCN solution of 0.02 mM **2,** 1.1 mM $Me_{10}Fc$, 15 mM TFA, and 1.1 mM H_2O_2 at 25 °C under a N_2 atmosphere. Right: Time profile (absorbance at 780 nm) for the formation of $Me_{10}Fc^+$.

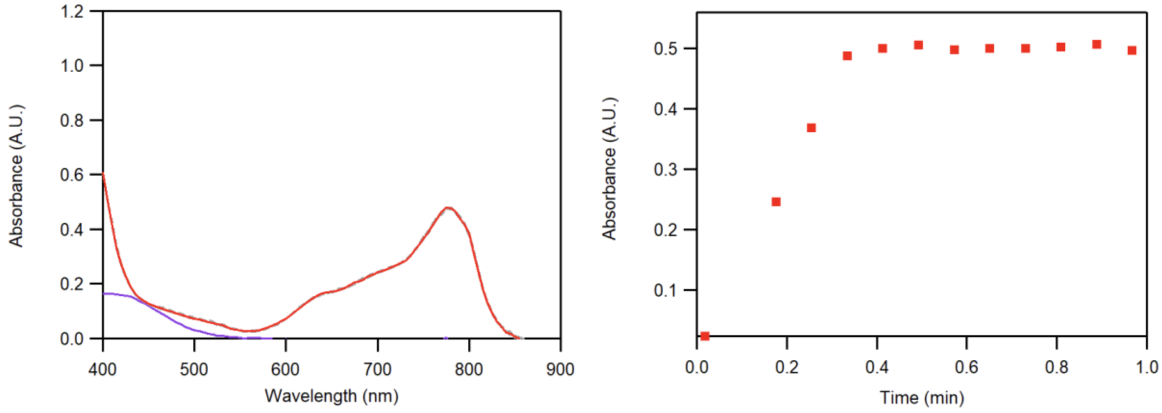

Figure S11. Left: Left: UV−Vis spectra of the formation of $Me_{10}Fc^+$ in a MeCN solution of 1.1 mM $Me_{10}Fc$, 15 mM TFA, and 1.1 mM H_2O_2 at 25 °C under a N_2 atmosphere. Right: Time profile (absorbance at 780 nm) for the formation of $Me_{10}Fc^+$.

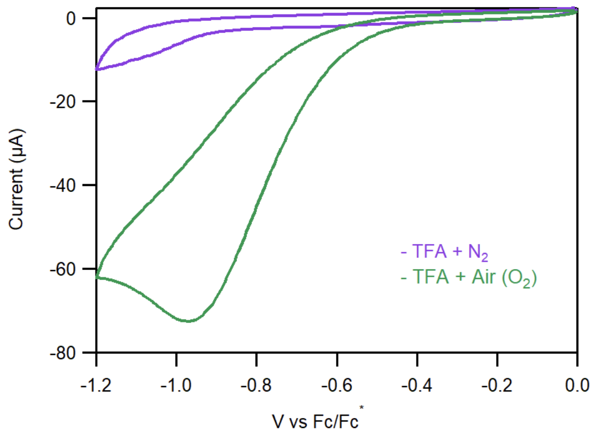

Figure S12. Background cyclic voltammograms of TFA (5 mM) under nitrogen (green) and under air (purple) in MeCN.

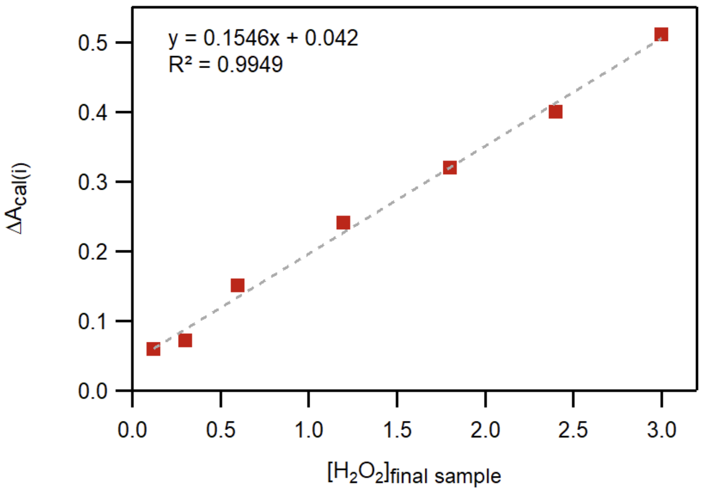

Figure S13. Plot of $\Delta A_{cal(i)}$ values against the concentration of H_2O_2 in the final sample solution ($[H_2O_2]_{final\ sample}$).

Table S1. Crystal data and structure refinement for $[Co(C_{24}H_{22}N_5O)][O_3SCF_3]$ (**1**).

Empirical formula	$C_{25}H_{22}CoF_3N_5O_4S$
Formula weight	604.46
Temperature	200(2) K
Wavelength	1.54178 Å
Crystal system	Monoclinic
Space group	$P2_1/c$
a/Å	15.1758(3)
b/Å	10.9022(2)
c/Å	15.5868(3)
α/°	90
β/°	92.3590(10)
γ/°	90
Volume/Å^3	2576.64(9)
Z	4
P_{calc} Mg/m^3	1.558
μ/mm^{-1}	6.552
F(000)	1236
Crystal size/mm^3	0.220 x 0.102 x 0.031
2Θ range for data collection/°	2.914 to 70.254
Index ranges	$-18 \leq h \leq 17$, $-11 \leq k \leq 12$, $-18 \leq l \leq 18$
Reflections collected	21060
Independent reflections	4686 [R(int) = 0.0614]
Completeness to theta = 66.000°	98.4 %
Absorption correction	Multi-scan
Max. and min. transmission	0.7533 and 0.4071
Refinement method	Full-matrix least-squares on F^2
Data / restraints / parameters	4686 / 0 / 357
Goodness-of-fit on F^2	1.028
Final R indexes [I>=2σ (I)]	$R_1 = 0.0541$, $wR_2 = 0.1433$
Final R indexes [all data]	$R_1 = 0.0610$, $wR_2 = 0.1508$
Extinction coefficient	0.00077(16)
Largest diff. peak/hole / e Å^{-3}	1.053 / -0.434

Table S2. Crystal data and structure refinement for $[Na][Co(C_{23}H_{21}N_6O)][O_3SCF_3]_2$ (**2**).

Empirical formula	$C_{25}H_2CoF_6N_6NaO_7S_2$
Formula weight	777.52
Temperature	200(2) K
Wavelength	1.54178 Å
Crystal system	Triclinic
Space group	P-1
a/Å	12.0195(2)
b/Å	12.6206(2)
c/Å	12.9509(2)
α/°	108.6800(10)
β/°	115.5750(10)
γ/°	101.9320(10)
Volume/Å^3	1535.81(5)
Z	2
P_{calc} Mg/m^3	1.681
μ/mm^{-1}	6.628
F(000)	786
Crystal size/mm^3	0.240 x 0.090 x 0.040
2Θ range for data collection/°	4.202 to 70.293
Index ranges	$-14 \leq h \leq 14$, $-15 \leq k \leq 15$, $-15 \leq l \leq 14$
Reflections collected	19492
Independent reflections	5398 [R(int) = 0.0472]
Completeness to theta = 66.000°	96.0 %
Absorption correction	Multi-scan
Max. and min. transmission	0.7534 and 0.3833
Refinement method	Full-matrix least-squares on F^2
Data / restraints / parameters	5398 / 0 / 434
Goodness-of-fit on F^2	1.080
Final R indexes [I>=2σ (I)]	R_1 = 0.0442, wR_2 = 0.1130
Final R indexes [all data]	R_1 = 0.0456, wR_2 = 0.1145
Extinction coefficient	0.0051(4)
Largest diff. peak/hole / e Å^{-3}	0.691 and -0.457
